# Practical Guidelines for Standardised Resolution of Important Protocol Deviations in Clinical Trials Conducted in Sub-Saharan Africa

**DOI:** 10.1007/s43441-023-00604-3

**Published:** 2024-01-29

**Authors:** Armel Zemsi, Lorraine Jinette Guedem Nekame, Nuredin Mohammed, Elizabeth Stanley Batchilly, Edgard Dabira, Sheikh Omar Sillah, Gibbi Sey, Daisy H. Williams, Bai-Lamin Dondeh, Carla Cerami, Ed Clarke, Umberto D’Alessandro

**Affiliations:** 1 MRCG at LSHTM, Atlantic Boulevard, Fajara, P.O. Box 273, Banjul, The Gambia; 2 Elizabeth Glaser Pediatric AIDS Foundation (EGPAF), Yaoundé, Cameroon

**Keywords:** Guideline, Clinical trial, Protocol deviation, Monitoring

## Abstract

A clinical trial is any research on human subjects that involves an investigational medicinal product or device. Investigational medicinal products include unlicensed drugs or drugs used outside the product license (e.g. for a new indication) (ICH-GCP). As per the internationally accepted ICH-GCP guidelines, clinical trials should be conducted strictly per the approved protocol. However, during the lifecycle of a trial, protocol deviations may occur. Under ICH efficacy guidelines, protocol deviations are divided into non-important (minor) or important (major), and the latter can jeopardise the participant’s rights, safety or the quality of data generated by the study. Existing guidelines on protocol deviation management do not detail or standardise actions to be taken for participants, investigational products, data or samples as part of a holistic management of important protocol deviations. Herein, we propose guidelines to address the current literature gap and promote the standardisation of actions to address important protocol deviations in clinical trials. The advised actions should complement the existing local institutional review board and national regulatory authority requirements.

## Introduction

The International Council for Harmonisation (ICH) guidance on Good Clinical Practice (GCP) provides a unified standard for the conduct of clinical trials and participants’ protection. It contributes to regulatory authorities’ worldwide acceptance of clinical trial data [1]. Complying with the approved study protocol is a key principle of this guidance. Any departure from the approved trial protocol is a protocol deviation. Protocol deviations may be considered important (major) or non-important (minor). The important deviations can significantly affect key study data's completeness, accuracy, and reliability or affect participant’s rights, safety, or well-being. Categorising a deviation as important depends on the trial design, the nature of the study data, the subject protections described in the protocol and the planned analyses [2]. However, a comprehensive listing of possible deviations in a trial has been developed, indicating for each of them whether they are important (major) or non-important (minor) [3].

Over the last two decades, the number of clinical trials conducted worldwide has been rising, with the sharpest increases occurring in the sub-Saharan African (sSA) countries [4, 5], despite the skills and technical gaps of the national regulatory agencies (NRA) of this region. For instance, according to the World Health Organization (WHO) global benchmarking tool, which defines the maturity levels of regulatory authority, most countries in sSA lack mature NRAs. These NRAs cannot perform the core regulatory functions, which include providing quality trial oversight [6, 7]. In addition, most sSA NRAs are not part of the various ICH bodies, with only the South African and the Nigerian NRAs being observers and no sSA NRA being a member[8]. Nonetheless, the availability of ICH-GCP guidelines and the derived additional clinical trials guidelines suitable for the challenges of the sSA context, such as WHO-GCP and the African Vaccine Regulatory Forum (AVAREF) tools, provide additional guidance to investigators, sponsors, institutional review boards (IRBs) and NRAs on clinical trials oversight and implementation in this specific setting [9, 10]

All clinical trial guidelines identify regular monitoring as a critical quality assurance activity as it permits early detection of protocol deviations and implementation of preventive and corrective actions to minimise their cost on the quality of the study [11]. Existing guidelines provide practical guidance and best practices for various monitoring approaches, including onsite or remote monitoring and exhaustive or risk-based and statistical monitoring that will allow the identification of protocol deviations and their management [3, 12–17].

However, there remains a need for specific guidelines to manage participants, investigational products, study data, and samples in the event of an important protocol deviation. Here, we propose guidelines with the following aims: (1) standardise the resolution of important deviations across settings, institutions, and therapeutic areas; (2) aid inexperienced institutional review boards and national regulatory authorities; (3) provide better guidance to trial monitors and sponsors; (4) facilitate the reporting of important protocol deviations and their management in clinical trial manuscripts.

## Materials and Methods

These guidelines have been developed through a collaborative panel of clinical trials’ subject matters experts, including principal investigators, regulatory experts, clinical research associates, data managers, and statisticians, who have extensive experience in implementing clinical trials and collaborating with regulatory institutions, academic institutions, industry, and clinical research organisations in sSA countries.

The panel included a total of 12 experts:

- 3 Principal investigators (PIs) with over 15 years of experience designing and implementing academic and industry-sponsored clinical trials in West Africa. Two of these investigators have the academic rank of professor and have extensive expertise in vaccine trials ranging from phase 1 to phase 4, targeting meningitis, yellow fever, measles, rubella and malaria in paediatric and adult populations. One of the investigators also has experience in controlled human infection model (CHIM) trials, drug trials for various infectious diseases and mass drug administration trials. The third investigator specialises in designing and implementing nutritional trials investigating nutritional supplements to curve down iron deficiency anaemias in children, pregnant women, and adults. All the investigators routinely sit on national ethics and scientific committees, supporting the ethical and scientific reviews of trials to be implemented in West Africa.
- 1 Biostatistician with the academic rank of associate professor and over 10 years of contribution in clinical trials design, in building randomisation utilities and statistical monitoring algorithms suitable for resources-limited settings. The member also has extensive expertise in analysing datasets, elaborating the clinical study reports (CSR) and contributing as a member to independent data monitoring committees (IDMC) of trials implemented in West Africa.
- 1 Data manager with expertise in building trial databases architecture and electronic data capture tools, which are CFR 21 part 11 compliant, suitable for deployment in resource-limited settings and integrating real-time quality control mechanisms of data collected.
- 3 research governance and trial regulations experts with extensive experience interacting with NRAs of West and Central African countries and providing sponsor-type oversight to clinical trials implemented in these regions. They also routinely sit on national ethics and scientific committees in West Africa.
- 2 Clinical research associates and 2 clinical trial managers with long experience conducting independent monitoring of clinical trials, managing and coordinating clinical trials across West Africa and providing GCP training to research teams in West, Central, and East Africa.

These experts reviewed a comprehensive list of important protocol deviations. They agreed on the best actions for the participants, the investigational product, the study data, and the study samples.

## Results

The detailed guidelines for each important deviation are presented in Table 1. These deviations were organised into 6 main categories: (1) Deviations with the informed consent, (2) deviations during participant’s screening and inclusion, (3) deviations during randomisation, blinding, and investigational product administration, (4) deviations during participants’ follow-up, safety and trial procedures, (5) deviations during transport and storage of Investigational products and samples, (6) deviations with equipment calibration.

**Table 1:**
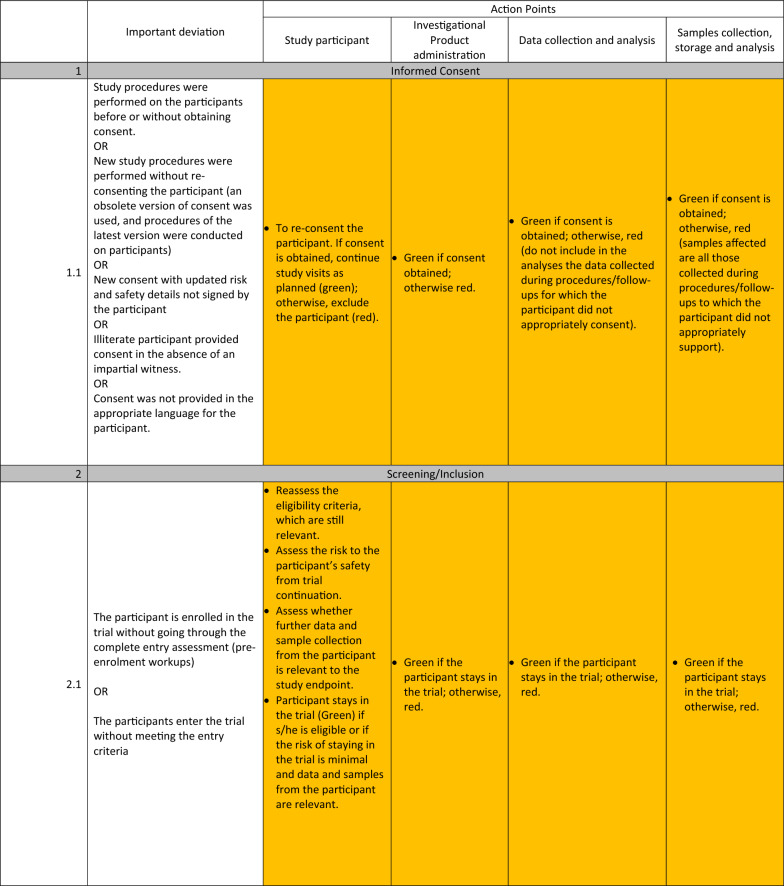

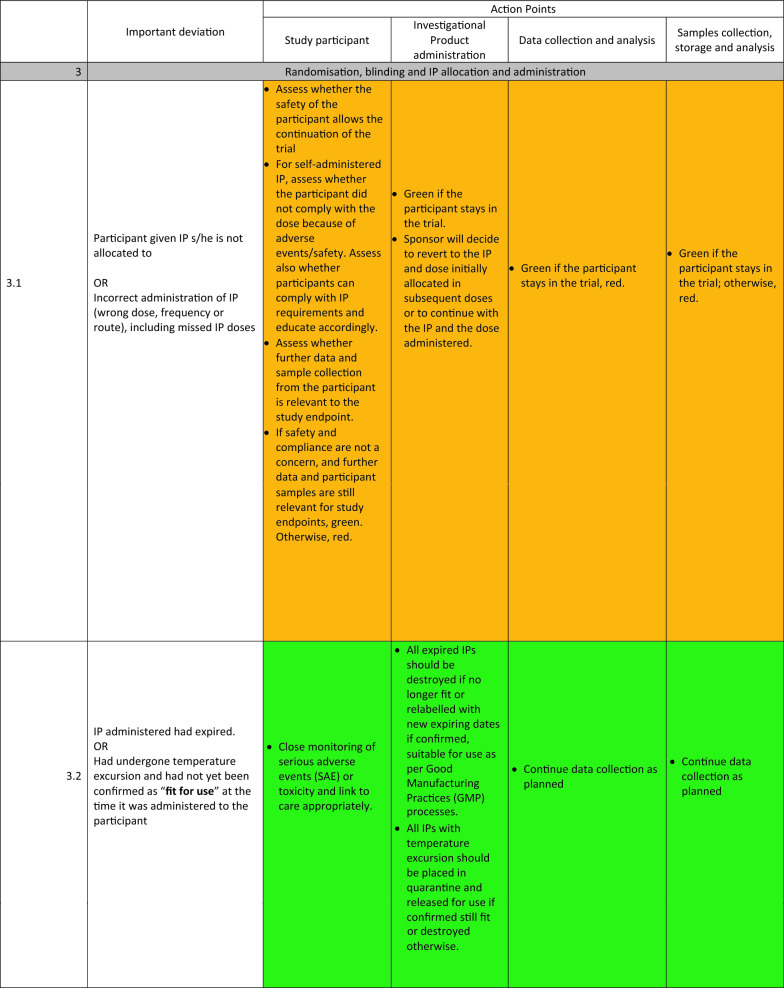

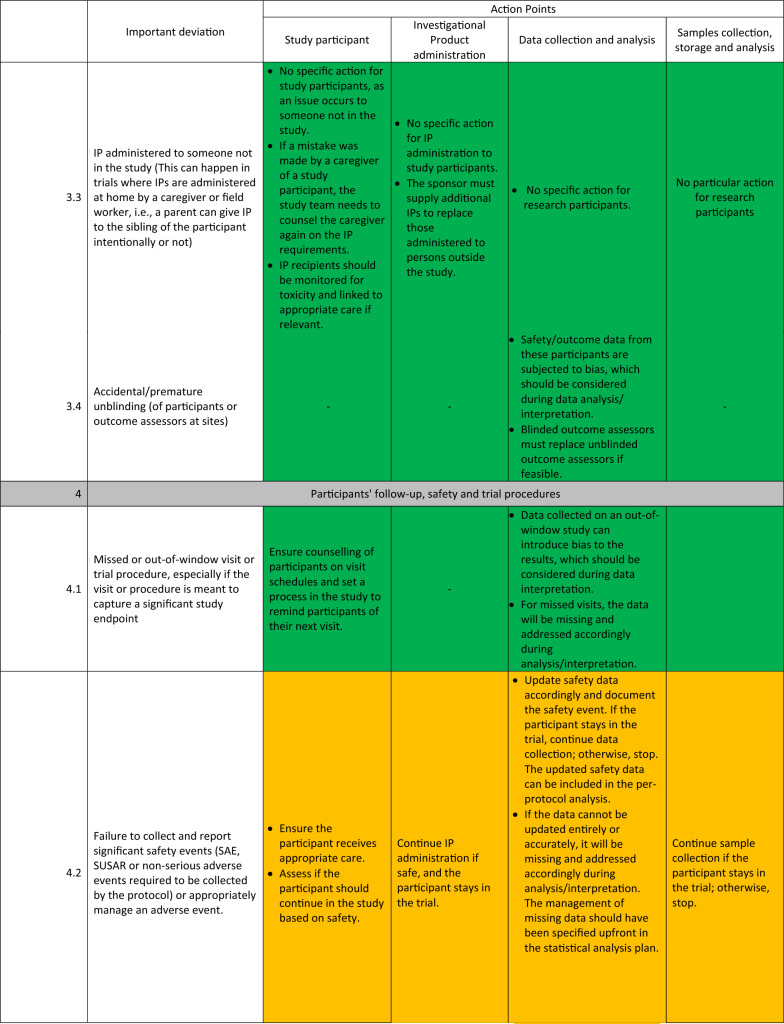

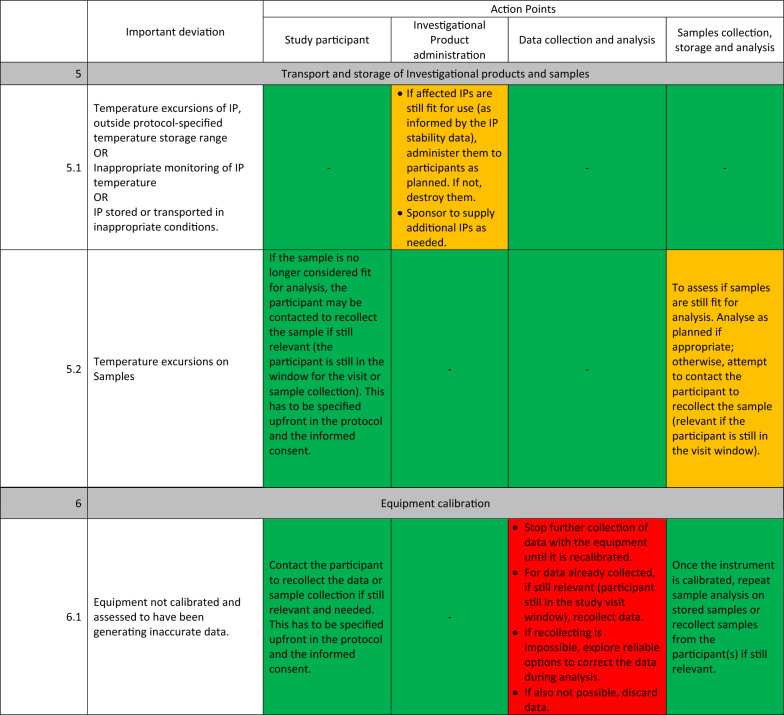
Standardized actions to take for each important deviation.

The experts identified three main types of actions to take once an important deviation has occurred:

- STOP (represented with red colour): These actions consist of discontinuing the participant affected by the deviation from the trial while still monitoring safety events as relevant, stopping further administration of the investigational product to the participant, and stopping additional data and sample collection from the participant.
- CONTINUE (represented with green colour): The trial procedures will continue as planned in this type of action. If relevant, the participant’s trial visit affected by the deviation can be repeated, the IP readministered, and the data and sample recollected.
- REASSESS (represented with orange): Here, additional parameters of the important deviation will be reassessed. Based on the reassessment outcome, the STOP or CONTINUE group of actions will be recommended. These additional parameters are related to the participant’s safety and desire to continue the study and the usefulness of the data and sample affected for the study endpoints.

The actions in these guidelines are designed to preserve study participants’ safety and rights and study and data integrity. They are also intended to limit the exclusion of the participants from the trial due to important protocol deviations but tend to be inclusive. These are the minimum actions to address holistically important deviations in clinical trials. Local regulations might require additional steps.

## Discussion

Protocol deviations can occur in all clinical trials conducted. Inadequate consent forms and failure to follow the investigational plan are the main findings observed in clinical trials inspected by the Food and Drug Administration (FDA) of the USA, accounting, respectively, for 28% and 34% of all the deficiencies observed over 30 years (July 1977 to December 2009)[18].

### Informed Consent

The informed consent originates in the Nuremberg code and has evolved. It is an essential step in clinical trials, during which sufficient information is provided to potential participants so they can voluntarily decide to participate in the study [19]. Informed consent is key to ensuring that the participants are aware of their rights and that these are preserved. Failure to appropriately consent participants will be an important deviation, as it will result in the participants being subjected to a procedure or to risk they did not understand or agree to.

The risk of performing trial procedures on a participant without appropriate consent is likely higher in resource-limited settings. In these settings, consent is faced with many challenges, including language barriers, level of education, false expectations from the participant towards the trial, and religious and community influence [19–21]. These challenges will result in insufficient comprehension when joining the study and in future drop-outs. Many studies have attempted to establish the best approaches to consenting and the optimal amount of information to provide, resulting in better comprehension by participants from various social and educational backgrounds [22–25]. Though there has not been a single best approach identified, these studies provide valuable information to sponsors and sites in deciding the most appropriate consent approach to be used in their specific study setting.

In these guidelines, for important deviations related to the informed consent, we recommend to re-consent the participant appropriately, and if the participant is still willing to continue in the study, to proceed with the trial activities as planned. If the participant declines to be re-consented, we recommend that the team discontinues the participant from the trial and does not process or analyse the samples and data collected during the procedures to which the participant did not consent to. This recommendation might not be applicable if the deviation is identified when the participant has completed the trial, has dropped out of the trial or is not reachable by the study team. In this case scenario, the management of the data and sample collected from this participant will be best decided by the IRB.

### Screening and Inclusion

After the screening process, participants are included only if they meet all the inclusion criteria and have none of the exclusion criteria. Enrolling participants who do not meet key eligibility criteria may compromise the study’s safety and scientific value and be considered an important protocol deviation. However, in some instances, the deviation from the eligibility criteria is intentional when the screening values of the participant are out of the accepted screening ranges but still borderline. At the study design stage, ensuring flexibility within the protocol without negatively affecting the participant’s safety or the study’s scientific value will contribute to limiting these types of deviations [26]. We advise that in case of important deviations to screening and inclusion, the team maintains the participant in the trial if the risk to the participant in continuing the trial is minimal and the data and samples collected from the participant are still relevant to answer the study questions. It is however crucial to limit the enrolment of ineligible participants into trials and in the era of electronic data capture tools, systems can be built to automatically flag at the enrolment stage participants whose screening values do not meet the study requirement and disallow data collection from them.

### Randomization, IP Administration and Trial Follow-Up Procedures

Deviations to randomisation, IP administration plans, and trial follow-up procedures required to assess primary trial endpoints are important deviations because they can significantly affect participant’s safety, well-being, and the reliability of key study data. Though these deviations can virtually occur in every trial, complex study designs, multiple study endpoints, the number of procedures per visit and the number of investigational sites have been positively associated and shown to be predictive of high incidence of protocol deviation in trials [27].

In these guidelines, we recommend that participants’ data are always considered for analysis despite deviation. Statistical methods exist to analyse participants’ data collected after deviations have occurred. Where the outcome data are available, it is commonly recommended to use intention-to-treat analysis. This pragmatic approach includes all participants as part of the study arm they were randomly assigned, regardless of the treatment they received or their compliance to treatment when these deviations result in missing data, as in the case of a missed trial visit or study procedure, statistical methods to infer these data, such as multiple imputations have been well elaborated [28].

### Transport, Storage of Investigational Products and Samples and Equipment Calibration

Investigational products and samples collected in trials might require controlled temperature ranges for transport and storage. Some samples are required to be processed within a specific timeline from their collection. Limited infrastructure and logistics are predictable challenges to proper transportation and storage of IPs and samples. Careful assessment of the needs is required to identify areas requiring strengthening and upgrade. Before the trial starts, sponsors can address these challenges by carefully assessing the sites’ needs and upgrading the infrastructure and capacity as necessary.

Equipment not calibrated or maintained can be considered a significant noncompliance if the equipment is used to measure a key study endpoint or to store an investigational product. Local availability of expertise is essential for equipment that requires frequent calibration or preventive maintenance.

### Protocol Deviations Management

Existing literature describes the processes and toolkits to implement in clinical trials to facilitate detection, review and analysis, reporting and close-out of protocol deviations [3, 11, 29]. Prevention, detection, documentation and trend analysis are critical in managing protocol deviations in clinical trials.

Prevention starts at the inception of the study and continues throughout the end. At the study design stage, involving persons with the necessary operational experience who will carefully evaluate the context and ensure that the protocol suits the chosen study setting will be crucial for the sponsor in preventing future deviations [30]. Additionally, adequate training of staff and setting up quality control and assurance mechanisms for all clinical trial procedures will reduce the incidence of protocol deviations.

Documenting protocol deviations and the actions taken to address them and prevent their recurrence is also critical, as it allows notification of various stakeholders on the deviation and allows monitoring of the progress towards resolution of deviations in trials.

With the advent of electronic tools in clinical trials, such as electronic data capture tools, it is now possible to integrate quality control steps during data collection, which will prevent deviations, integrate automatic detection of deviations in trial procedures and to speed up paperless detection, documentation and reporting of protocol deviations.

Performing root cause assessments and trend analyses of important protocol deviations will guide the research team in identifying shortcomings in the trial and establish whether protocol amendments, team retraining or replacement, or site closure are necessary to prevent important deviations from recurrence.

## Conclusion

With these guidelines, we aimed to provide specific guidance for participants, investigational products, samples and data analysis in the context of important protocol deviations. We recommend actions aligned with any existing guidance from the local ethical or regulatory authority overseeing the trials for each aspect.

The nature of the actions proposed in these guidelines will depend on whether the participant affected by the important protocol deviation is still consenting to stay in the trial, if administering the investigational product is still safe for the participant, and if collecting samples and data from the participant is still relevant and will contribute to answering the clinical trial’s research question. When an important deviation occurs, the investigative team should inform and agree with the sponsor and the relevant institutional review board to implement these actions.

Standardisation in clinical trials promotes credibility and acceptability of results. However, while standardised guidelines for conducting clinical trials exist, there is no standardised approach to managing important protocol deviations. This paper provided standardised practical actions for the participants, the investigational product, the study data and the samples for each type of important deviation. These actions are either to stop procedures or participation in the trial, reassess additional factors to determine the action to take or continue trial procedures.

## Data Availability

Not applicable.
